# KRT5^high^ TP63-expressing urothelial basal cells act as a driver to bladder urothelium regeneration in rabbit

**DOI:** 10.1186/s13287-025-04417-z

**Published:** 2025-06-07

**Authors:** Jiasheng Chen, Mingming Yu, Lin Wang, Hua Xie, Yiqing Lv, Yichen Huang, Yue Hong, Fang Chen

**Affiliations:** 1 Department of Urology, School of Medicine, Shanghai Children’s Hospital, Shanghai Jiao Tong University, 355 Luding Road, Shanghai, 200062 PR China; 2 Department of Urology, Shanghai Sixth People’s Hospital, Shanghai Jiao Tong University School of Medicine, Shanghai, 200233 PR China; 3 Shanghai Eastern Urological Reconstruction and Repair Institute, Shanghai, 200233 PR China; 4 Department of Ultrasound in Medicine, Shanghai Sixth People’s Hospital, Shanghai Jiao Tong University School of Medicine, Shanghai, 200233 PR China; 5 Stem Cell Center, Shanghai Sixth People’s Hospital, Shanghai Jiao Tong University School of Medicine, Shanghai, 200233 PR China; 6 School of Life and Health Sciences, Hainan Province Key Laboratory of One Health, Collaborative Innovation Center of Life and Health, Hainan University, Haikou, 570228 PR China

**Keywords:** Bladder urothelium regeneration, Urothelial basal cell, Tissue engineering, Cell sheet, Capsule vascular bed

## Abstract

**Background:**

Urothelial regeneration is a crucial part of bladder tissue engineering. However, there is a lack of ideal “seed cells” in current practices. Here, we demonstrated that a sub-population of p63 positive basal cells could be activated and differentiate into intermediate and superficial umbrella cells after full-thickness mucosal resection in rabbit.

**Methods:**

A focal mucosal resection model was used to characterize the role of different urothelial cells during regeneration. Urothelial basal cells were isolated from rabbit bladder mucosa and cultured in vitro. The basal cells were then transplanted in vivo in a manner of cell sheet for reconstruction.

**Results:**

Via single-cell RNA sequencing (scRNA-seq), it has been confirmed that the cluster of KRT5^high^ TP63-expressing cells possesses a ‘‘stemness’’ signature which can give rise to lineage cell types sequentially. With a strong support from the underneath pre-set capsule vascular bed, the transplanted cell sheet could develop into a physio-morphology resembled to the native mucosa in vivo. Importantly, we validated that the bioengineered urothelium implemented perfect barrier function after implanted to bladder.

**Conclusions:**

In summary, bioengineering urothelium with KRT5^high^ TP63-expressing basal cells on a capsule vascular bed offers a promising strategy for bladder tissue engineering and provides a model for drug screening and bladder disease research.

**Supplementary Information:**

The online version contains supplementary material available at 10.1186/s13287-025-04417-z.

## Introduction

The bladder lumen is lined by urothelium, a transitional epithelium consisting of three cell types: superficial umbrella cell, intermediate cell and basal cell [1]. The primary role of urothelium is to generate a robust barrier to protect underlying tissue from toxic substances within hypertonic urine. Bladder reconstruction is desired in various clinical scenarios, such as bladder cancer, congenital malformations, neuropathic bladder, trauma, infection or inflammation [2]. Currently, gastrointestinal segments remain the primary source of material for bladder reconstruction, however, the transposed intestine segments maintain an absorptive and mucus-producing epithelium after replacing the bladder, which lead to numerous clinical complications including metabolic disturbance, stone formation, chronic infections and secondary malignancies [3]. As the field of tissue engineering advances, bioengineered bladder is expected to replace gastrointestinal tissue as a new gold standard for bladder reconstruction. Although the generation of a bioengineered tissue with a morphology similar to that of the native bladder has already been accomplished, the restoration of a functional bladder using this strategy has not been achieved so far, which is hindering the application of bioengineered bladder in clinical settings. Urothelial regeneration is a crucial part of bladder tissue engineering as any leakage of urine into the underlying tissues may provoke a fibrotic outcome associated with randomly organized collagen fibres, muscle contraction, and compromised bladder capacity [4]. Thus, to establish a strategy enabling efficient bladder full-thickness reconstruction with functional urothelium is of clinical importance, particularly in cases requiring to construct a neobladder.

Despite differentiated urothelial cells obtained from biopsies being used for the generation of bioengineered urothelium with barrier function, these cells present limited capacity for proliferation and differentiation in vitro [5–8]. Mesenchymal stem cells (MSCs) derived from bone marrow or adipose tissue have been explored towards urothelial cell differentiation, however, none of them displayed urothelial-oriented potential and the regenerated tissue generally lacked typical urothelium markers [9, 10]. Although induced pluripotent stem cells (iPSCs) and embryonic stem cells (ESCs) can differentiate into functional urothelial cells, considerations of preparation efficiency, tumorigenic risk and ethical issues restrict its wide application [11, 12].

Normally, the adult urothelium is quiescent with a turnover rate of 3–6 months, whereas urothelium shows rapid proliferation and differentiation in response to damage, and the protective barrier is soon restored [13, 14]. Several lines of evidence support the existence of dedicated stem/progenitor cell populations in adult bladder urothelium that mediate its renewal during normal homeostasis and repair after injury [15]. In adult rodent, label-retaining study demonstrated that slow-cycling label-retaining cells (LRCs) located in the basal layer of the bladder urothelium may be the putative stem/progenitor cells [16, 17], but another study indicated that uropathogenic Escherichia coli (UPEC) inoculation induced the proliferation of LRCs within both basal cell and intermediate cell layers [18]. Furthermore, lineage tracing studies indicated that Krt5 positive basal cell and uroplakin (Upk) positive intermediate cell both or individual can serve as stem/progenitor cells engaging in urothelial regeneration after injury, which depending on the type or severity of injury [19–22]. Overall, these studies suggested a presence of tissue-specific stem/progenitor cells within bladder urothelium whilst their location and true identity remain controversial. To this end, if the putative stem/progenitor cells can be identified, isolated and expanded, it would be a new option for bioengineered urothelium.

In the current work, to assess the feasibility and provide pre-clinical evidence, we applied rabbit for experiment rather than rodent, reaching a better correlation to clinical significance due to the regeneration size. Via histological methodology and 5-bromo-2-deoxyuridine (BrdU) labeling, we evidently demonstrated that Krt5 positive basal cells, serving as stem/progenitor cells, can be activated and differentiate into intermediate and superficial umbrella cells after full-thickness mucosal resection in a rabbit model. Then, single-cell RNA sequencing (scRNA-seq) data proved that the cluster of KRT5^high^ TP63-expressing cells with a ‘‘stemness’’ signature can give rise to intermediate and umbrella populations sequentially in normal and mucosa injured conditions. Most importantly, for the first time we successfully isolated and expanded KRT5^high^ TP63-expressing basal cells in vitro and explored the feasibility of autologous KRT5^high^ TP63-expressing basal cells transplantation for urothelial regeneration.

## Materials and methods

### Experimental animals

Adult New Zealand rabbits weighing 2.5–3.0 kg were obtained from the Animal Laboratory of the Shanghai Sixth People’s Hospital and raised under clean conditions with separate cage. Immunodeficient NOD-PrkdcscidIl2rgem1/Smoc (NSG) mice were provided by Shanghai Model Organisms. All animal experimental procedures were conducted under IACUC guidelines and approved by the IACUC committee of Shanghai Children’s Hospital. The rabbits were anesthetized with an intravenous injection of 20–30 mg/kg sodium pentobarbital before surgery and euthanized with an overdose of pentobarbital when needed. Obvious complications such as infection, wound disruption or poor healing at any point throughout the whole process were criteria for exclusion. The work has been reported in line with the ARRIVE guidelines 2.0.

### Generation of focal mucosal resection model

Rabbits were anesthetized with 2% pentobarbital sodium (30 mg/kg, Sigma), and a low midline laparotomy incision was made to expose the bladder. The bladder dome was opened and four 5 − 0 polypropylene (Prolene^®^, Ethicon Inc.) nonresorbable marking sutures were placed into the posterior of bladder wall to demarcate a 2 × 2 cm wound site. Incisions were performed around the wound region, then the mucosa was resected from the bladder surface (Fig. S1). Bladder was closed in one layer using 5 − 0 polyglactin continuous sutures. An 8 Fr transurethral polyurethane catheter was placed, and the wound were closed in layers using a routine method. Rabbits were euthanized at 2 days, 1 week, 2 weeks, and 4 weeks post operation. Tissue specimens from the injured region of bladder were subjected to histological staining analyses as detailed below.

### BrdU incorporation

For short term BrdU label-retaining experiment, 6 adult New Zealand rabbits were injured as described above and injected intraperitoneally with BrdU (Sigma) 50 mg/kg body weight at 2 days post-surgery, then 3 of them were sacrificed 6 h after and the other 3 were euthanized at 1 week post-surgery. For long term BrdU label-retaining experiment, 6 adult New Zealand rabbits were injected intraperitoneally with BrdU 50 mg/kg body weight per day for 7 days. Thereafter 3 of them were euthanized 4 months later and the other 3 were injured as described, which were killed another 4 weeks after. Tissue specimens from normal and injured region of bladder were subjected to histological staining analyses.

### Histological staining

All tissue specimens were fixed with 4% paraformaldehyde and paraffin-embedded. Serial sections (5 μm) were cut in the center of the tissue along the longest axis and evaluated by hematoxylin-eosin (H&E) and immunofluorescence (IF) staining. For IF staining, the sections were heated in citrate sodium antigen retrieval buffer for antigen retrieval and labeled with the primary antibodies as listed below: mouse anti-p63 (Abcam, ab735), rabbit anti-Krt5 (Abcam, ab193894), rabbit anti-Krt20 (Abcam, ab76126), rabbit antiKi67 (Abcam, ab15580) and rat anti-BrdU (Abcam, ab6326), mouse anti-Upk3a (Santa Cruz, sc-166808), and mouse anti-Zo-1 (Invitrogen, 33-9100). They were then incubated with secondary antibodies and counterstained with 4’6-diamidino-2-phenylindole (DAPI). The slides were viewed and photographed using Leica DM6 upright digital microscope (Leica Application Suite X software) and the entire IF images were captured using NanoZoomer S210 Digital slide scanner.

### Rabbit urothelial cells isolation and KRT5^high^ TP63-expressing basal cells culture

Rabbit was anesthetized and bladder was exposed as aforementioned. A small opening was cut into the exposed muscle layer and bluntly separated from its underlying mucosa layer with a curved clamp, then a 2.0 × 2.0 cm area of mucosa was extracted from bladder. The bladder was closed in one layer using 5 − 0 polyglactin continuous sutures. Next, the mucosa was finely cut into 1mm^3^ pieces with blade and digested with dissociation buffer (Ham’s F12, 1 mg/ml collagenase II (Gibco), 100 µg/ml Pen/Strep) for 1–2 h at 37 °C with gentle rocking. Dissociated cells were washed thoroughly in cold wash buffer (Ham’s F12, 5% fetal bovine serum, 100 µg/ml Pen/Strep), and plated on irradiated 3T3-J2 feeder layers in 6-well culture plate (3 wells/sample), followed by 2–3 weeks culture as previously described [23], in a 7.5% CO2 atmosphere at 37 °C. The morphology of cultured KRT5^high^ TP63-expressing basal cells was observed by a phase-contrast microscope, and microphotographs were taken at 100-fold magnification. The colonies were fixed in 4% paraformaldehyde and stained with rhodamine-B (Sigma). For IF staining, cells were cultured on glass cover slips in 6-well plates and fixed with 4% paraformaldehyde. After blocking with 10% Goat serum, the cells were labeled with the primary antibodies followed by incubating with secondary antibodies and counterstained with DAPI. The slides were viewed and photographed as above described.

### KRT5^high^ TP63-expressing basal cells xenograft assay

KRT5^high^ TP63-expressing basal cells cultured for 3 weeks were harvested using TrypLE, and resuspended in Dulbecco’s Modified Eagle’s Medium. Prior to subcutaneous injection, the cell suspension was further mixed 1:1 (v/v) with growth factor reduced Matrigel and then transplanted onto NSG mice with a total volume of 250 µl/injection. Animals were euthanized at 4 weeks post-transplantation and the subcutaneous implant was harvested for paraffin sectioning followed by histological staining.

### RNA-seq and analysis

Purified KRT5^high^ TP63-expressing basal cells were harvested from culture via differential trypsinization to ensure complete clearance of feeder contaminant. Total RNA was extracted from cultured KRT5^high^ TP63-expressing basal cells using a RNeasy Mini Kit (Qiagen) and treated with DNase I. Sequencing libraries were generated using NEBNext Ultra RNA Library Prep Kit for Illumina (NEB, USA). Sequencing was performed by Jiayin Biotechnology Co., Ltd (Shanghai, China). RNA-Seq libraries were sequenced on an Illumina Novaseq 6000 platform and 150 bp pairend reads were generated. Reads Counts for each gene in each sample were counted by HTSeqv0.6.0, and FPKM (Fragments Per Kilobase Millon Mapped Reads) and then were calculated to estimate the expression level of genes in each sample. Heatmap for relative expression of selected gene was generated with R package.

### scRNA-seq and analysis

The single cell suspensions from normal and injured bladder tissues were prepared as aforementioned. scRNA-seq were performed by Jiayin Biotechnology Ltd. (Shanghai, China), according to instruction manual of the Chromium Single Cell 3’ Reagent Kits v3. Cellular suspensions were loaded on the Chromiu Controller (10x Genomics, Pleasanton) to generate Gel Bead-In-Emulsions (GEMs). Barcoded sequencing libraries were conducted following the instruction manual of the Chromium Single Cell 3’ Reagent Kits v3 (10x Genomics). Following the library preparation, the sequencing was performed with paired-end sequencing of 150nt each end on one lane of NovaSeq 6000 per sample. The reads were mapped onto the rabbit genome using a standard CellRanger pipeline. A raw unique molecular identifier (UMI) count matrix was generated after Cell Ranger processing, then the matrix was converted into a Seurat object by R package Seurat. Low-quality cells with UMI numbers < 500, gene numbers < 200, or mitochondrial genes > 15% were filtered and the rest of cells were retained for downstream analysis.

Cell-clustering and sub-clustering analyses were performed with the FindClusters function offered by Seurat with proper resolutions. UMAP was used to display identified cell clusters and sub-clusters. The cell clusters were annotated based on the expression of the canonical marker genes and referring genes from previously reported study. The R package ClusterProfiler was used for GO and KEGG analysis of the differential marker genes among subclusters. Pseudotime analysis of epithelial cells was conducted using Monocle2 pipeline to determine the dramatic developmental trajectory and translational relationships.

### Generation of capsule vascular bed

Tissue expander capsule was performed as previously described [24–26]. Briefly, skin incisions were cut in the right inguinal region, and the inguinal fat pad surrounded superficial circumflex iliac (SCI) artery and vein was carefully isolated to expose SCI vessels. A sterile, 15 ml spherical expander was placed close to the SCI vessels underneath the bilateral inguinal skin. After wound closure, the expander was injected with 5 ml of saline intraoperatively and another 5 ml at 5 and 7 days post operation to achieve the final volume of 15 ml. Another 1 week later, the tissue expander was removed and the vascularized capsules was harvested for histological staining or prepared for cell sheet transplantation.

### Autologous KRT5^high^ TP63-expressing basal cell sheets transplantation

Rabbit urothelial cells were isolated as aforementioned and seeded onto 6-well temperature-responsive culture plates (Thermo Fisher Scientific) in the presence of the 3T3 feeder-layers. After cocultured for 3 weeks, the culture temperature was reduced to 20–25 °C for 5–6 min to obtain intact KRT5^high^ TP63-expressing basal cell sheet. The KRT5^high^ TP63-expressing basal cell sheet was fixed and paraffin-embedded for histological staining according to the above method. For autologous KRT5^high^ TP63-expressing basal cell sheets transplantation, these sheets were transferred onto the capsule vascular bed, which was induced 2 weeks ago in the same rabbit. The transplanted cell sheets were covered with 0.3 mm-thick silicone membranes to prevent adhesion and vascularization from the upper skin. Rabbits were euthanized at 1 week, and 3 weeks after transplantation. The grafts were resected and fixed to histological staining analyses.

### Implantation of vascularized capsule to bladder

Nine rabbits were randomly divided into 3 groups. Three of them only received capsule flaps as Capsule only group and the others received capsule flaps pre-planted with KRT5^high^ TP63-expressing basal cell sheets as Capsule with cells group. In addition, another 3 sham-operated rabbits as Control group. Only the authors in charge of research design and conduct of the treatment were aware of the group allocation. Rabbit was anesthetized and bladder was exposed as aforementioned. An incision of 3 cm was made in the bladder far from the biopsy site. The vascularized capsule or capsule with KRT5^high^ TP63-expressing basal cells (3 weeks after transplantation) was isolated on its SCI vascular pedicle. The pedicle capsule flap was pulled from the groin space to the abdominal cavity through a small hole in the abdominal wall, and was sutured into the bladder defect using 5 − 0 polyglactin continuous sutures. In the control group the bladder was closed in one layer. An 8 Fr transurethral polyurethane catheter was placed, and the wound was closed in layers using a routine method. Prophylactic cefuroxime sodium was administered intravenously for 5 days (0.5 g/day).

### Dynamic contrast-enhanced magnetic resonance imaging (CE-MRI)

Four weeks after implantation of vascularized capsule to bladder, rabbit was anesthetized. The bladder was catheterized with an 8 Fr transurethral polyurethane catheter to drain the urine from the bladder and Gd-DTPA solution (0.2 mmol Gd/kg diluted to 30 mL in saline) was administered intravesically through the catheter. MRI scanning was conducted on a GE Discovery 450 3.0 T 70 cm wide bore clinical MRI system. MRI scans were obtained before Gd-DTPA instillation and 5, 15 and 30 min post-contrast. A total of five regions-of-interest (ROIs) were taken along the periphery of augmented segments from each bladder crosssection displayed on Paravision (v 5.0, Bruker Biospin); and five corresponding ROIs were taken from sham-operated intact bladders. MRI signal intensities were calculated by subtracting post-instillation from pre-instillation (background) signals from the ROIs for each animal.

### Statistical analyses

#### Statistical validation of scRNA-seq clustering and annotations

Raw UMI counts were normalized using the Seurat LogNormalize method, scaling features to 10,000 reads per cell. The top 2,000 highly variable genes were identified using the vst method in Seurat to reduce dimensionality. Data integration across biological replicates (normal and injured conditions) was performed using the Seurat integration pipeline (CCA anchor-based alignment) to mitigate batch effects. Principal Component Analysis (PCA) was applied, and the top 30 principal components (PCs) were selected based on the elbow plot of explained variance. Cells were clustered using the Louvain algorithm with a resolution parameter of 0.8 (optimized via the clustree R package to avoid over-/under-clustering). Differentially expressed genes (DEGs) for each cluster were identified using the Wilcoxon rank-sum test (implemented in Seurat’s FindAllMarkers function) with thresholds of adjusted *p*-value < 0.01 (Benjamini-Hochberg correction) and log2(fold change) > 0.5. Clusters were annotated by cross-referencing DEGs with established urothelial markers (e.g., UPK3B/KRT20 for umbrella cells, KRT5/TP63 for basal cells). Enriched biological pathways for each cluster were computed via ClusterProfiler using a q-value cutoff of 0.05.

#### Pseudotime analysis validation

Pseudotime trajectories were constructed using Monocle2 with DDRTree dimensionality reduction. Genes used for trajectory inference were selected based on differential expression testing (q-value < 0.01) across clusters. Branch-dependent gene expression changes were validated using Monocle2’s BEAM (Branch Expression Analysis Modeling) with a q-value < 0.001. To ensure trajectory reliability, we performed bootstrapping (1,000 iterations) and confirmed that the pseudotemporal ordering was consistent across subsampled datasets.

#### Statistical methods for general analyses

DEGs between cultured KRT5high TP63-expressing cells and in vivo basal cells were identified using DESeq2 with an adjusted *p*-value < 0.05 and|log2(fold change)| > 1. Signal intensities were compared across groups using one-way ANOVA followed by Tukey’s HSD post hoc test (adjusted *p* < 0.05). Data are presented as mean ± SD.

## Results

### Krt5 positive basal cells with stem/progenitor cells potential contributed to urothelial regeneration following focal mucosal resection in rabbit bladder

**Fig. 1:**
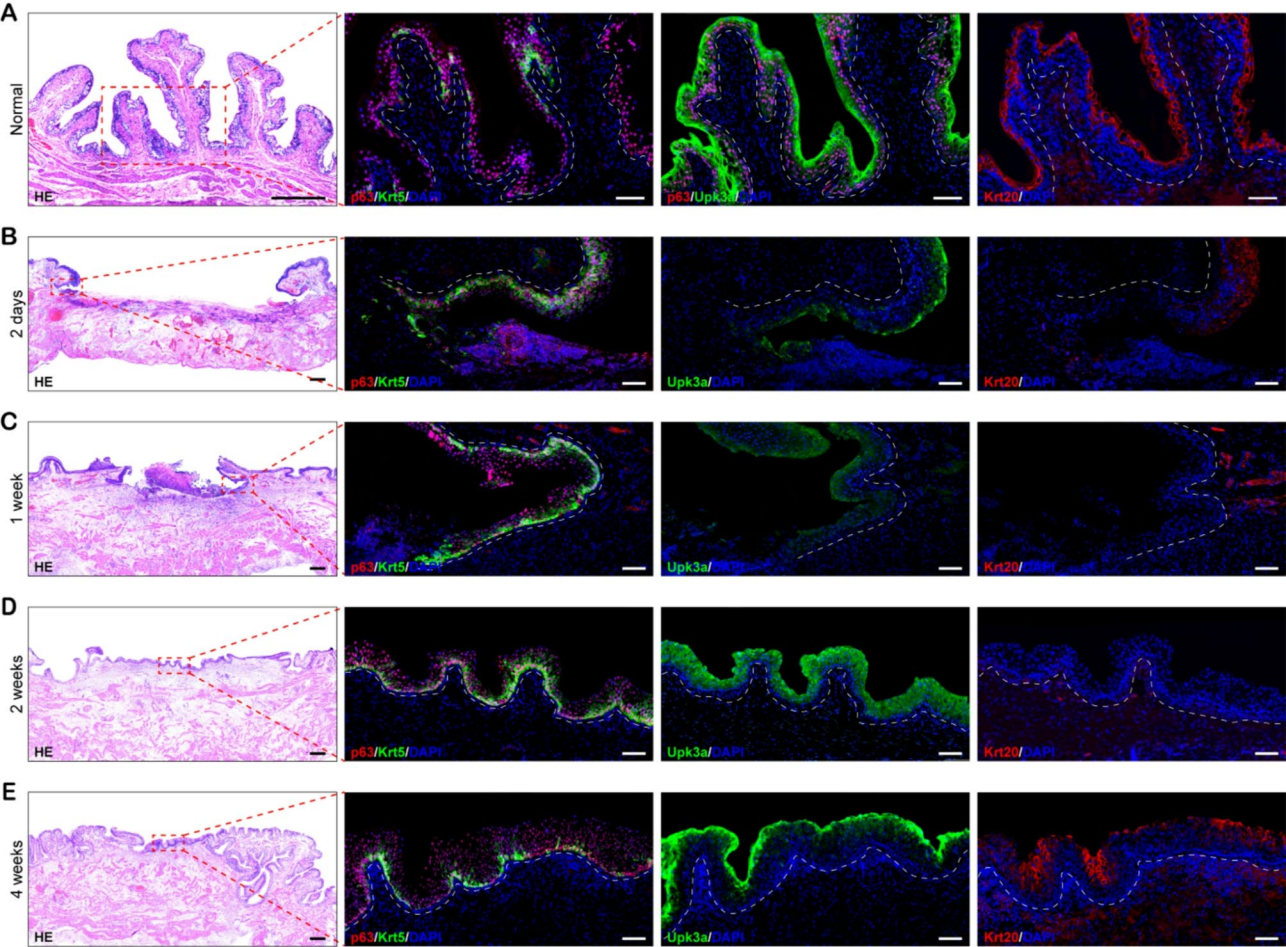
Phases of urothelial regeneration at regions of focal mucosal resection. H&E staining and IF staining of normal (**A**) and focal mucosa removed bladder wall after 2 days (**B**), 1 week (**C**), 2 weeks (**D**), 4 weeks (**E**). White dashed lines indicate the epithelial-mesenchymal boundary. Scale bar: 500 μm (H&E staining), 100 μm (IF staining)

Umbrella, intermediate, and basal cell populations of the bladder urothelium have been previously distinguished according to combinatorial markers [13, 19–22]. Based on these characterizations, we performed immunostaining for Krt5, Upk3a, p63, and Krt20 in rabbit bladder specimens to discriminate urothelial subpopulations. Histological examination confirmed that the rabbit bladder urothelium, in consistent with murine urothelium, comprises basal, intermediate, and umbrella cell populations according to cell morphology, location and the expression of protein markers (Fig. 1A), whereas there were more layers of intermediate cells in rabbit. Unlike murine basal cells with homogeneous expression of Krt5 protein (Fig. S2-3), Krt5 immunoreactive cells dispersedly distributed in rabbit basal cell layer, and all Krt5 positive basal cells coexpression with p63 but not vice versa (Fig. 1A), indicating that there are heterogeneous subpopulations in rabbit basal cells.

**Fig. 2:**
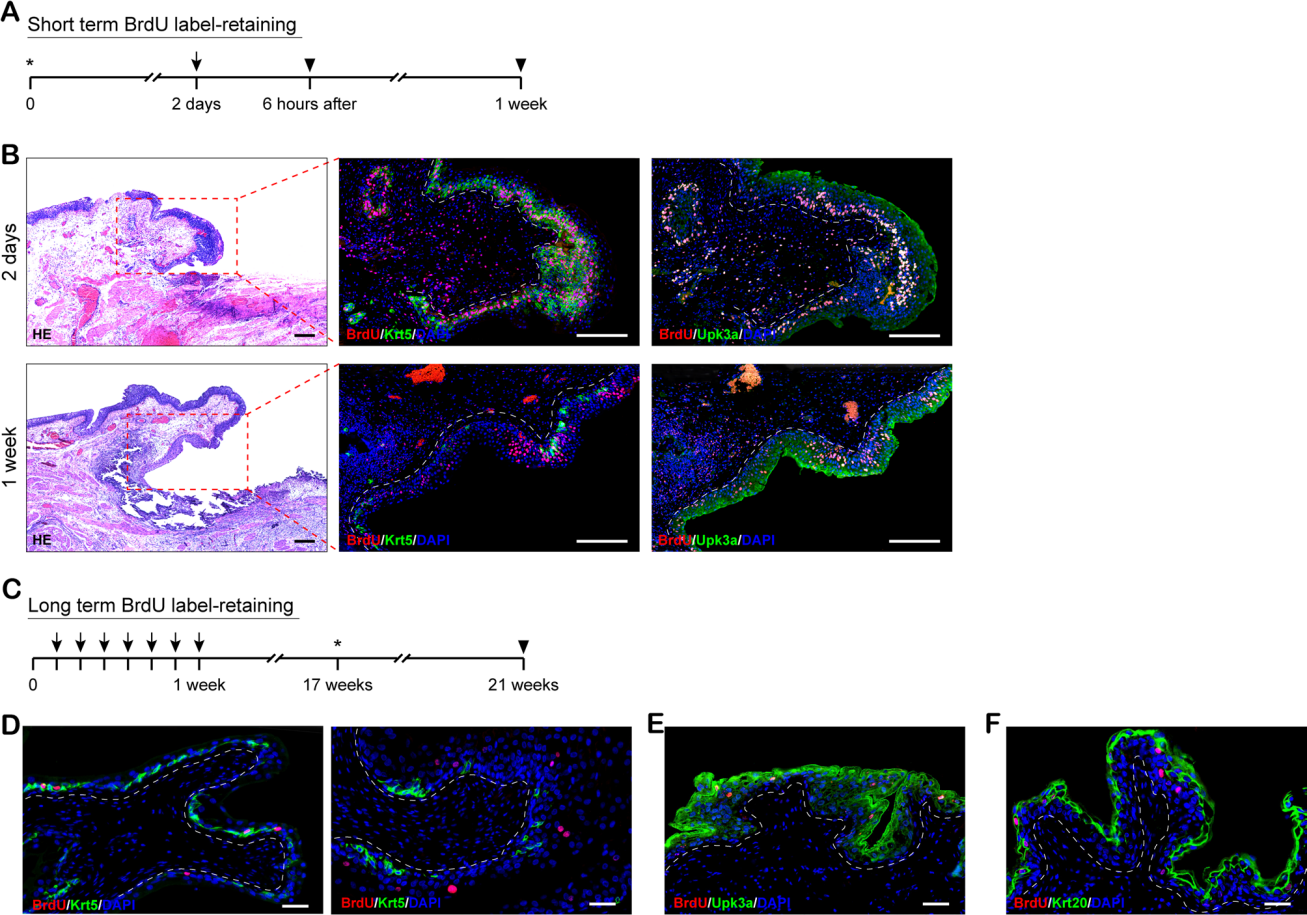
BrdU label-retaining study of bladder urothelial cells during urothelial regeneration. (**A**) The timeline of short-term BrdU label-retaining experiment. Asterisks indicate inducing of mucosa injury. Arrows indicate BrdU injections. Arrowheads indicate sacrifice of animals. (**B**) In short term BrdU label-retaining experiment, H&E (left) and IF staining of BrdU, Krt5 (middle) and BrdU, Upk3a (right) in the margin of injured region at 2 days and 1 week after mucosa injury. (**C**) The timeline of long-term BrdU label-retaining experiment. Asterisks indicate inducing of mucosa injury. Arrows indicate BrdU injections. Arrowheads indicate sacrifice of animals. (**D**) In long term BrdU label-retaining experiment, IF staining of BrdU and Krt5 in the normal bladder urothelium at 4 months after BrdU withdrawal (left) and in the regeneration region of injured urothelium at 1 week after mucosa injury (right). (**E**) In long term BrdU label-retaining experiment, IF staining of BrdU and Upk3a in the regeneration region of injured urothelium at 1 week after mucosa injury. (**F**) In long term BrdU label-retaining experiment, IF staining of BrdU and Krt20 in the regeneration region of injured urothelium at 1 week after mucosa injury. White dashed lines indicate the epithelial-mesenchymal boundary. Scale bar: 250 μm (B), 50 μm (D-F)

**Fig. 3:**
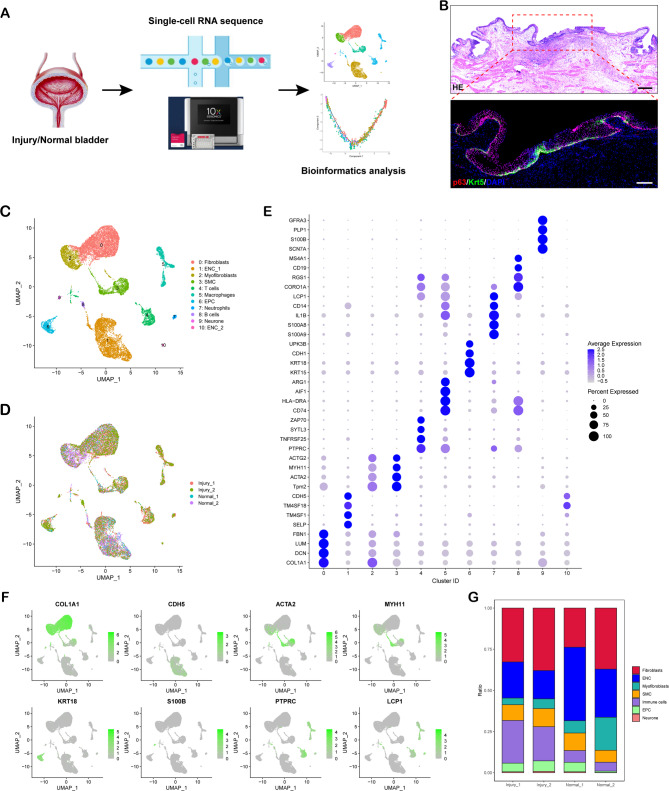
scRNA-Seq analysis of all cells in rabbit bladder wall under normal and injured conditions. (**A**) Schematic diagram of experimental design for scRNA-seq. (**B**) H&E and IF staining for injured mucosa showing a region equivalent to those used for scRNA-Seq. White dashed lines indicate the epithelial-mesenchymal boundary. Scale bar: 500 μm (H&E), 200 μm (IF). (**C**) UMAP visualization of all cells from bladder urothelium under normal and injured conditions and cells are colored by clusters. (**D**) Cells are colored by replicate identity in the UMAP plot. (**E**) Dot plot shows marker genes in different clusters identified. (**F**) UMAP plot showing expression of the indicated genes in each cluster. (**G**) Bar plot shows the proportion of each cell type in the different samples

In order to characterize the role of different urothelial cells during regeneration upon mucosa injury, we established a focal mucosal resection model as previously reported [21]. A 2 × 2 cm wound was introduced in the posterior of bladder wall with full-thickness mucosa removal (Fig. S1) and the entire tissue that encompassed the injured region was harvested at different time points post-surgery for histological examination. In Fig. 1B, H&E staining showed that the full-thickness of mucosa were completely removed in the injured area, and the lamina propria with acute inflammation and edema exposed to luminal surface at 2 days after injury. With time forward, the inflammation and edema disappeared gradually and lamina propria was covered by de novo urothelium step by step (Fig. 1C-E). IF staining demonstrated that a large number of Krt5 positive cells distributed continuously around the wound and extended to the center of the wound bed after mucosa injury (Fig. 1B and C), which was consistent with the concept that basal cell involved in front edge movement is the key point of epidermal wound healing [27]. By 4 weeks after injury, the Krt5 positive cells presented as island distribution in basal layer like normal urothelium of rabbit bladder (Fig. 1E). Upk3a positive cells initially appeared above the front edge of wound at 1 week after injury (Fig. 1C), and the immunoreactivity for Upk3a was obvious in superficial cells by 2 weeks (Fig. 1D). Exhilaratingly, Krt5 positive cells protruded into the Upk3a positive cells and displayed overlapping expression of Upk3a at this stage of regeneration, indicating that these intermediate cells derived from Krt5 positive cells. Despite scarce Krt20 immunoreactive cell was observed until 2 weeks after injury (Fig. 1D), almost all superficial cells showed the immunoreactivity for Krt20 by 4 weeks, demonstrating replenishment of differentiated superficial umbrella cells (Fig. 1E).

BrdU is a thymidine-analogue that can be incorporated into the DNA sequence of dividing cells during mitotic and transferred to progeny with cell division. BrdU is generally applied to assess cell proliferation rate and label slow cycling cells, which retain the analogue for longer periods of time. Using BrdU label-retaining assay has identified potential epithelial stem/progenitor cells that are almost quiescent and seldom divide in skin, intestine, and esophagus [28–30]. In present study, the BrdU label-retaining study was conducted according to prior scheme with some modifications [30] (Fig. 2A and C). The result of short-term BrdU label-retaining study show that there were many LRCs around the injured site, the majority of which were Krt5 positive cells at 2 days after focal mucosal resection, suggesting this compartment was active after injury (Fig. 2B). However, at 1 week following injury, we found that LRCs were predominantly located within Upk3a positive cells. These results suggest that injury leads to rapid induction of Krt5 positive basal cells proliferation and then fuels regeneration of the injured area. For long-term BrdU label-retaining study (Fig. 2C), IF staining with BrdU demonstrated that LRCs were primarily positioned in the basal cells and hardly seen in superficial umbrella cells or intermediate cells in 4 months after BrdU withdrawal, additionally, most LRCs were co-localized with Krt5 positive cells (Fig. 2D, left). We then introduced a focal mucosal resection injury in pre-labeled rabbit with BrdU 4 months ago. Histological examination of bladder urothelium 4 weeks after injury revealed that LRCs had migrated from the basal layer to suprabasilar layer (Fig. 2D, right). Co-staining BrdU with Upk3a or Krt20 proved that LRCs can differentiate into superficial umbrella and intermediate cells during urothelial regeneration (Fig. 2E and F).

Taken together, these data implied that the basal cells of rabbit bladder urothelium were heterogeneous, among which a sub-population of p63 positive basal cells, Krt5 positive basal cells, can differentiate into intermediate and superficial umbrella cells, exhibiting a pronounced role in urothelial regeneration after injury.

### Single-cell transcriptomics reveals the cluster of KRT5^high^ TP63-expressing cells as stem/progenitor cells in normal and mucosa-injured conditions

To comprehensively explore the cellular heterogeneity and dynamics of bladder mucosa under homeostasis and injured conditions, we performed droplet-based scRNA-seq using the 10x Genomics Single Cell Solution on samples isolated from injured (Injury_1, Injury_2) and normal (Normal_1, Normal_2) rabbit bladder (Fig. 3A). The injured samples were taken at 10 days after the introduction of a focal mucosal resection, corresponding to a stage where the injured area was just completely covered by de novo urothelium (Fig. 3B). After quality control filtering (Fig. S4A), 11,029 (from two injured biological replicates) and 9,819 (from two normal biological replicates) cells were maintained, and their single-cell transcriptomes were used for downstream analyses.

We integrated cells from the two conditions using the IntegrateData function in Seurat to correct batch effect and identified 11 distinct clusters via uniform manifold approximation and projection (UMAP) dimensionality reduction analysis (Fig. 3C and D). According to the expression of the canonical marker genes and referring genes from previously published scRNA-seq datasets (Fig. 3E and F; Fig. S4B), we manually annotated the cell clusters into fibroblasts (expressing COL1A1, DCN, LUM and FBN1), endothelial cells (ENC, expressing SELP, TM4SF1, TM4SF18 and CDH5), myofibroblasts (expressing ACTA2, MYH11 and COL1A1), smooth muscle cells (SMC, expressing Tpm2, MYH11 and ACTG2), T cells (expressing PTPRC, TNFRSF25, SYTL3 and ZAP70), macrophages (expressing CD74, HLA-DRA, AIF1 and ARG1), epithelial cells (EPC, expressing KRT15, KRT18, CDH1 and UPK3B), neutrophils (expressing S100A9, S100A8, IL1B and CD14), B cells (expressing CORO1A, RGS1, CD19 and MS4A1), and neurone (expressing SCN7A, S100B, PLP1 and GFRA3). Of these cells, the average percentages of immune cells including T cells, macrophages, neutrophils and B cells significantly increased in the injured replicates compared to normal replicates (Fig. 3G; Fig. S4C).

**Fig. 4:**
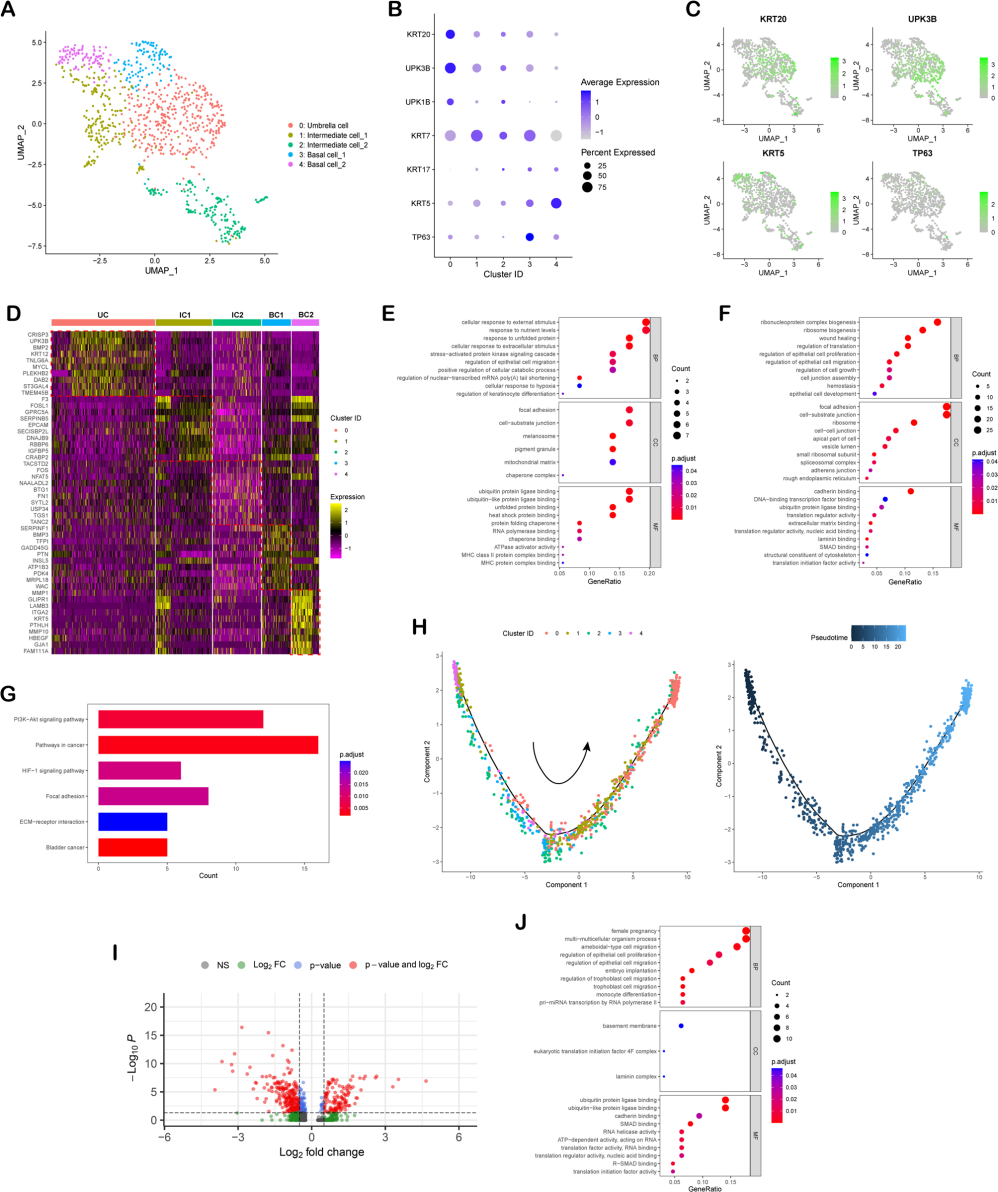
Subclustering epithelial cells shows the cell heterogeneity and lineage hierarchy of bladder urothelial cells. (**A**) UMAP visualization of sub-clustered urothelial cells, which colored by different cell types. (**B**) Dot plot shows marker genes in different clusters identified. (**C**) UMAP plot showing expression of the indicated genes in each cluster. (**D**) Heatmap for the top 10 differentially expressed genes in each cluster. Dotted lines outline differentially expressed genes. (**E**) GO analysis of upregulated genes in Basal cell_1 cells. (**F**) GO analysis of upregulated genes in Basal cell_2 cells. (**G**) Bar chart showing the significant KEGG terms of Basal cell_2 cells. (**H**) Pseudotime trajectory of urothelial cells. Cells are ordered from beginning (dark blue) to end (light blue) and colored by different cell types. (**I**) Volcano plot showing differentially expressed genes between Basal cell_2 cells derived from normal bladder and those derived from injured bladder. (**J**) GO analysis of upregulated genes in Basal cell_2 cells derived from injured bladder compared to those derived from normal bladder

To further analyze cellular heterogeneity of bladder urothelial cells, we selected all epithelial cells based on first-level clustering, and subjected them to a second round of cluster analysis. The epithelial cells could be classified as a total of 5 sub-clusters based on distinct marker panels (Fig. 4A). Cells of cluster 0 highly expressed the UPK gene family (UPK1B and UPK3B) and KRT20, were thus defined as Umbrella cell (UC) (Fig. 4B-D). Cells of cluster 1 and 2 exhibited moderate expression of UPK gene family, were referred to as Intermediate cell_1 (IC1) and Intermediate cell_2 (IC2). Of interest, these cells also expressed basal markers such as KRT5 and TP63 (Fig. 4B and C). The remaining cells of cluster 3 and 4 expressed KRT5 and TP63 but exhibited low levels of KRT20 or UPKs, and Gene Ontology Enrichment (GO) analysis of cluster 3 and 4 gene signatures supported that these two clusters were basal cells (Fig. 4E and F). Therefore, cluster 3 and 4 were named as Basal cell_1 (BC1) and Basal cell_2 (BC2) respectively. GO analysis revealed that the genes upregulated in Basal cell_1 cells comprised genes regulating cellular response to external stimulus, cellular response to nutrient levels, epithelial cell migration and regulation of keratinocyte differentiation, suggesting that Basal cell_1 may be a ‘transit amplifying’ subcluster that directly differentiate (Fig. 4E). For Basal cell_2 cells, GO enriched terms mainly related to ribosome biogenesis, regulation of epithelial cell proliferation and epithelial cell development, and enriched in PI3K-Akt and HIF-1 signaling pathway, which indicated that Basal cell_2 cells may be the stem/progenitor subcluster of bladder urothelium (Fig. 4F and G). It was obvious that the expression of KRT5 in Basal cell_2 cells was higher than that in Basal cell_1 cells (Fig. 4B-D), thus we defined the stem/progenitor subcluster as KRT5^high^ TP63-expressing basal cells. In addition, we applied Monocle 2 to generate a pseudotime trajectory plot and conclude the states among these urothelial cells (Fig. 4H). As expected, Basal cell_2 cells were placed at the beginning, and then transited to Basal cell_1 cells and Intermediate cells, while Umbrella cells expressing the terminal differentiation gene KRT20 at the trajectory terminus. Moreover, comparing to Basal cell_2 cells derived from normal bladder, those derived from injured bladder revealed distinct gene signatures (Fig. 4I), and GO enriched terms of the upregulated genes indicated that Basal cell_2 cells were predicted to be activated in injured condition (Fig. 4J).

Altogether, these single-cell transcriptome data supported the role of Basal cell_2 cells expressing TP63 and higher KRT5 as stem/progenitor cell of rabbit bladder urothelium in normal mucosa-injured conditions.

### Isolation and characterization of rabbit KRT5^high^ TP63-expressing basal cells in vitro

**Fig. 5:**
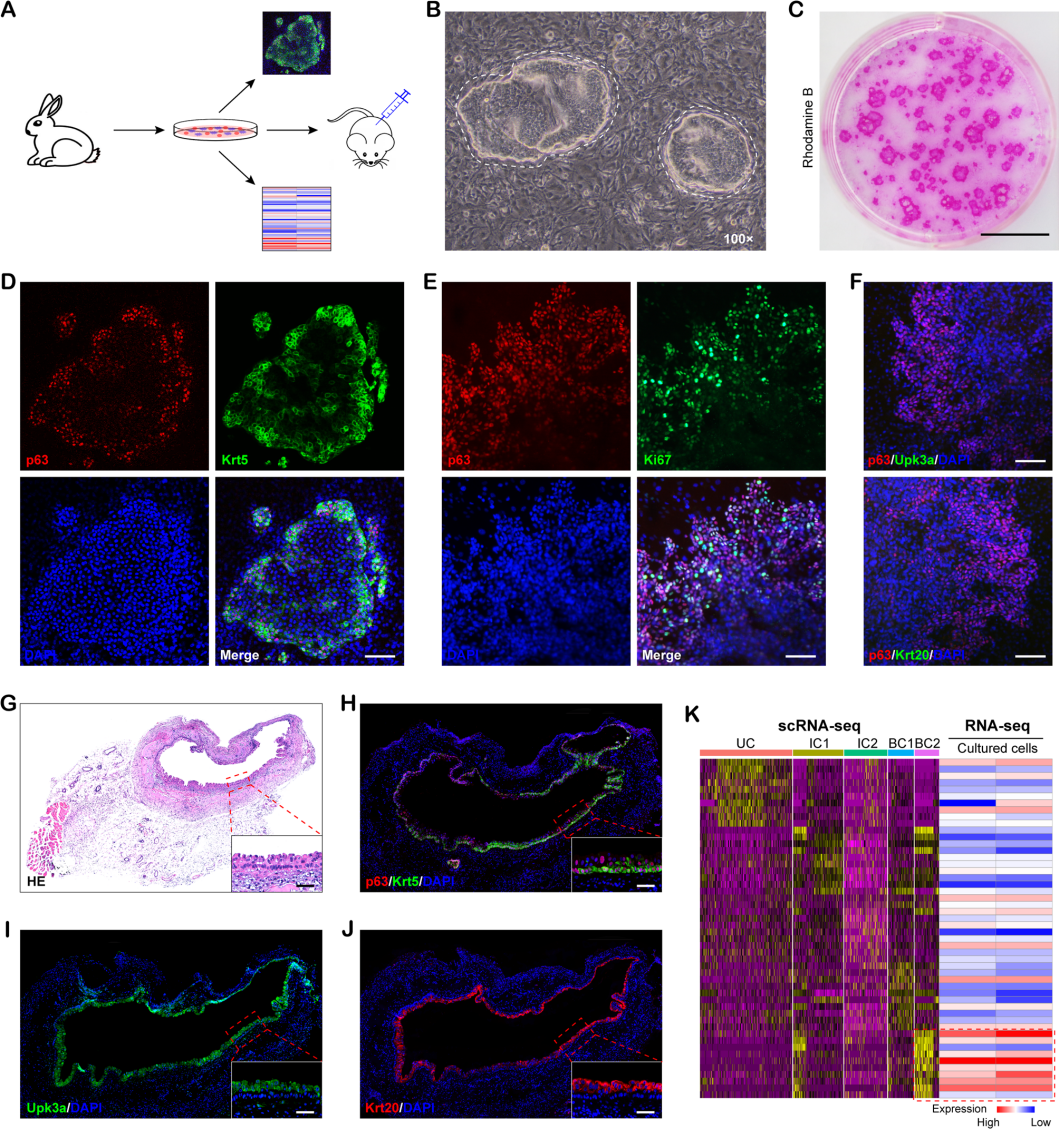
Isolation and characterization of rabbit KRT5^high^ TP63-expressing basal cells in vitro. (**A**) Schematic diagram depicting the process of experiment. (**B**) Phase contrast micrograph of colonies growing over irradiated feeder layer. Dotted lines indicate KRT5^high^ TP63-expressing basal cells colony. (**C**) Colony forming efficiency of KRT5^high^ TP63-expressing basal cells stained with Rhodamine B. (**D-F**) IF staining of colonies for the expression of p63 and Krt5 (**D**), Ki67 (**E**) and Upk3a and Krt20 (**F**). (**G**) H&E staining of xenografts derived from KRT5^high^ TP63-expressing basal cells. (**H-J**) IF staining of p63, Krt5, Upk3a and Krt20 on sequential xenograft sections. (**K**) Gene expression heatmap of 2 cultured KRT5^high^ TP63-expressing basal cells next to the cluster of KRT5^high^ TP63-expressing cells shown in Fig. 4D for ease of comparison. Red box highlights genes of interest. Scale bar: 1 cm (C), 100 μm (D-F), 50 μm (G-J)

In order to further examine the basal cell potential for bladder tissue engineering, we isolated and expanded KRT5^high^ TP63-expressing basal cells in vitro (Fig. 5A). Bladder urothelial cells were isolated from mucosal tissue, which was surgically extracted from rabbit bladder, and expanded on irradiated 3T3-J2 feeder layer supplemented with complete medium. Phase contrast photographs showed that the primary culture of urothelial cells began to form colonies within 1 week after seeding (Fig. 5B) and reached 70% confluency by 3 weeks (Fig. 5C), which suggested a good proliferative potential. By IF staining, the expanded cells were positive for the stem/progenitor cell markers p63 and Krt5 (Fig. 5D) and proliferation marker Ki67 (Fig. 5E), while negative for Upk3a and Krt20, markers for fully differentiated urothelium (Fig. 5F). Furthermore, to evaluate the differentiational potential of KRT5^high^ TP63-expressing basal cells, we transplanted the expanded KRT5^high^ TP63-expressing basal cells into immunodeficient mice subcutaneously, over 4 weeks, the histopathology results showed that the implanted KRT5^high^ TP63-expressing basal cells could form ‘‘micro-bladder’’-like structure, and confirmed by immunostaining with Upk3a and Krt20 (Fig. 5G-J). Moreover, the heatmap in Fig. 5K showed the gene expression of cultured KRT5^high^ TP63-expressing basal cells (next to the heatmap of scRNA-seq from Fig. 4D for comparison), which revealed a homogeneous expression profile in comparison to the cluster of KRT5^high^ TP63-expressing cells.

Collectively, the above findings demonstrated that we successfully generated KRT5^high^ TP63-expressing basal cells with the capability for self-renewal and differentiation in vitro.

### Generation of bioengineered KRT5^high^ TP63-expressing basal cell sheet that can resemble the native urothelium in vivo

**Fig. 6:**
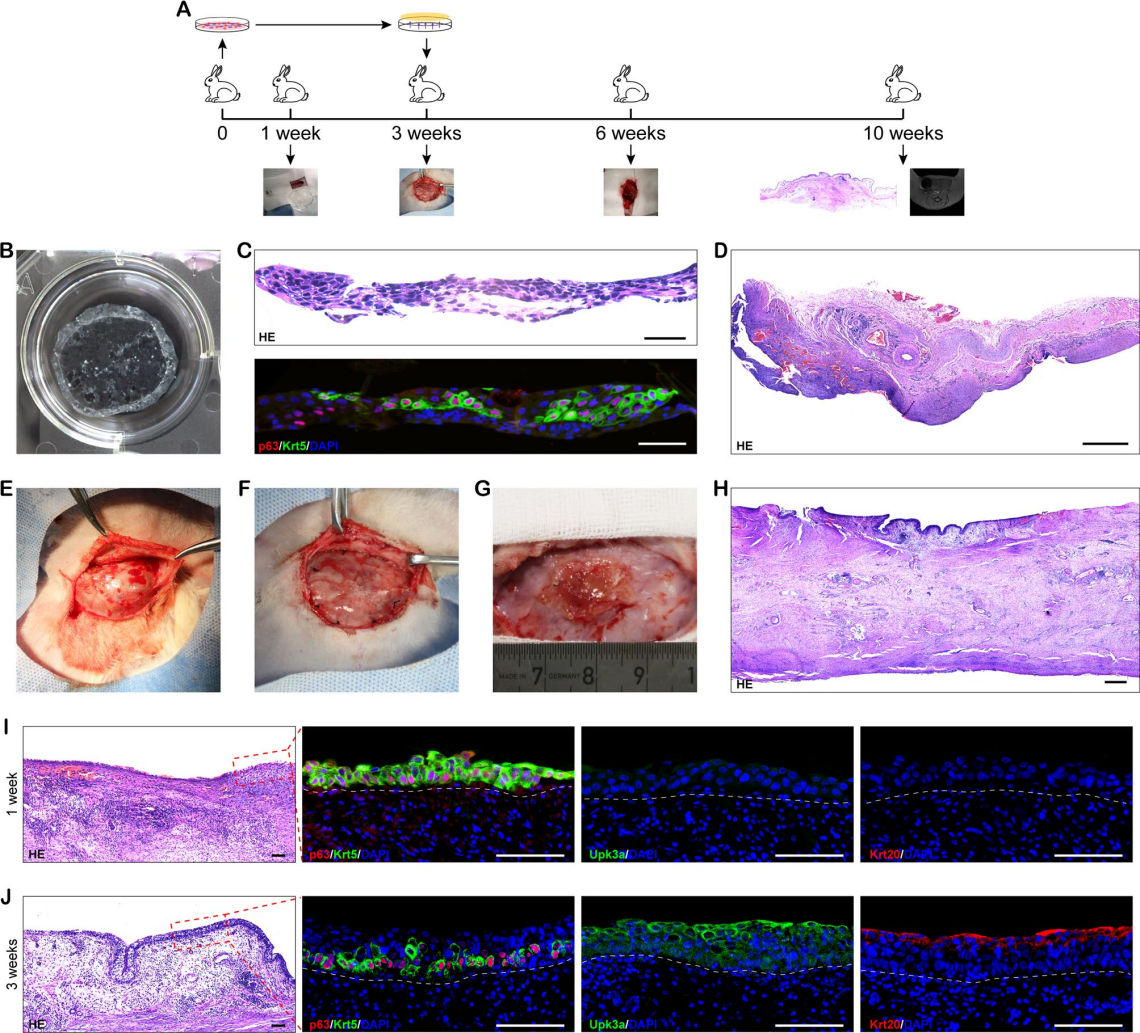
Bioengineered KRT5^high^ TP63-expressing basal cell sheet survived on the induced capsule vascular bed. (**A**) Scheme for the KRT5^high^ TP63-expressing basal cell sheet autografting experiment. (**B**) The gross appearance of a KRT5^high^ TP63-expressing basal cell sheet harvested by reducing temperature. (**C**) H&E and IF staining the cross-section of a KRT5^high^ TP63-expressing basal cell sheet. (**D**) H&E staining of the induced capsule. (**E**) Macroscopic view of the induced capsule vascular bed. (**F**) The KRT5^high^ TP63-expressing basal cell sheets were transplanted on the capsule vascular bed. (**G**) Macroscopic view of the transplanted KRT5^high^ TP63-expressing basal cells sheet on the capsule vascular bed for 1 weeks. (**H**) H&E staining of the capsule with transplanted cell sheets. (**I**,** J**) H&E staining and IF staining of the capsule with stacked KRT5^high^ TP63-expressing basal cells sheets at 1 week (I) and 3 weeks (J) after transplantation. White dashed lines indicate the sheet-capsule boundary. Scale bar: 100 μm (C), 1 mm (D, H), 100 μm (I, J)

To explore the feasibility of using autologous KRT5^high^ TP63-expressing basal cells for urothelium tissue engineering, we performed an experimental scheme (Fig. 6A) aiming to generate a functional vascularized mucosa tissue flap based on the “hospitable soil” of capsule vascular bed. Firstly, urothelial cells isolated from rabbit bladder were seeded on temperature-responsive culture dishes with irradiated 3T3-J2 feeder layer. After 3 weeks of culture, the bioengineered KRT5^high^ TP63-expressing basal cell sheet was harvested by temperature reduction as a single contiguous cell sheet, and IF staining showing the KRT5^high^ TP63-expressing basal cell markers as p63 and Krt5 (Fig. 6B and C). Subsequently, according to the manufacturer’s instruction, the in vitro pre-fabricated KRT5^high^ TP63-expressing basal cell sheets were transplanted onto the surface of capsule vascular bed (Fig. 6D-F), which was induced 2 weeks before. The vascular bed was generated by inserting a tissue expander around the SCI artery and vein from where numerous small vessels originated after injecting water into the expander, which we can observe in gross appearance and histopathology (Fig. 6D and E). After 1 week of KRT5^high^ TP63-expressing cell sheets transplantation, the macroscopical de novo tissues could be easily distinguished from the surrounding capsular tissue, however, the size of graft decreased (Fig. 6G) and histopathological staining demonstrated that a lawn of KRT5^high^ TP63-expressing basal cells confirmed by immunostaining with p63 and Krt5 resided on the surface of capsule (Fig. 6I). Over time, the de novo tissues increased in thickness reaching up to 4–5 cell layers at 3 weeks after transplantation (Fig. 6H and J), and resembled native urothelium morphologically, comprising different cell layers discriminated by relevant markers. These results suggested that bioengineered KRT5^high^ TP63-expressing basal cell sheet could survive on the surface of capsule and displayed a good capability of proliferation and differentiation.

**Fig. 7:**
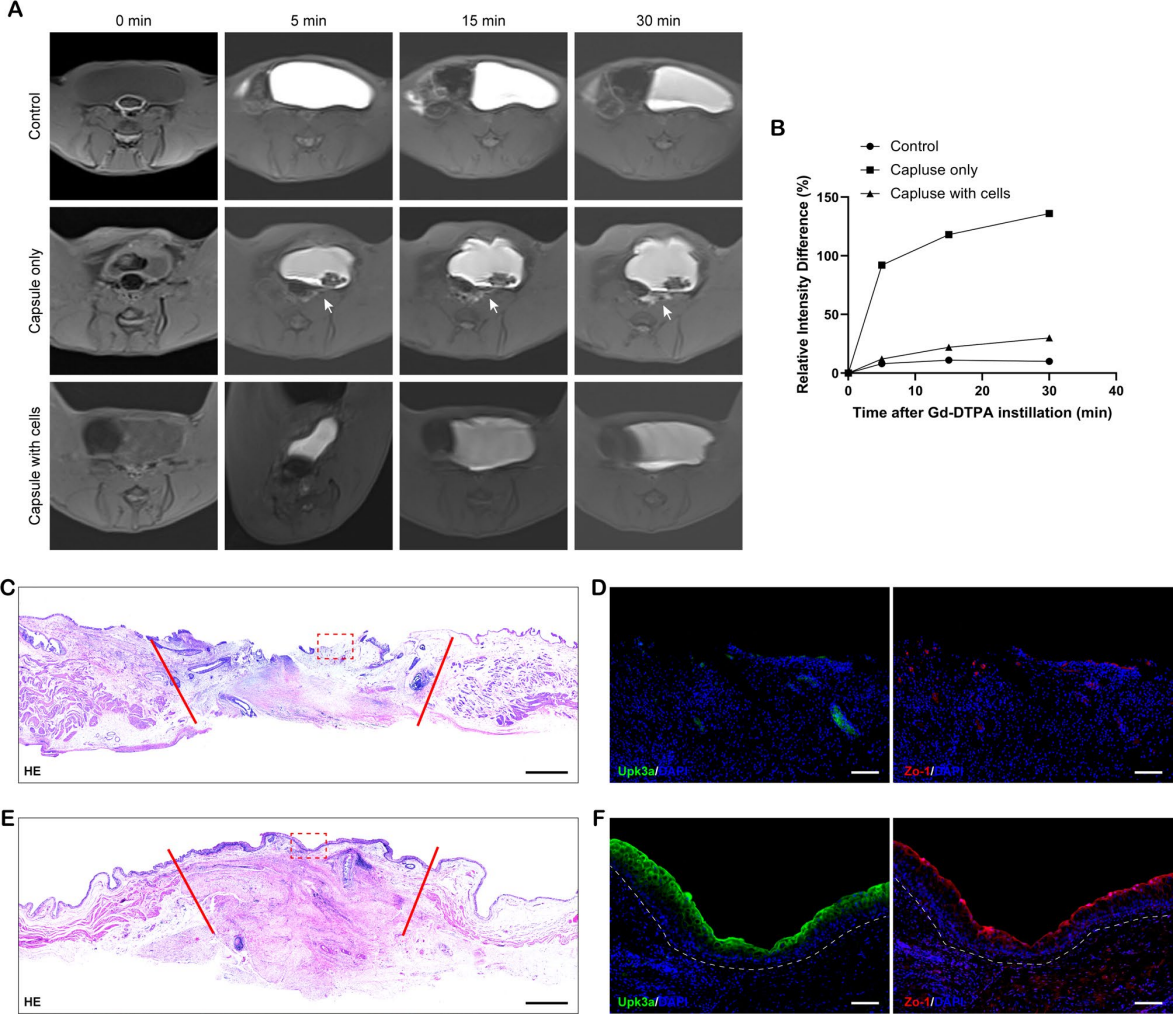
Permeability barrier function test of de novo urothelium from KRT5^high^ TP63-expressing basal cells. (**A**) CE-MRI visualization of Gd-DTPA signal from different groups. MRI was obtained before (0 min), and 5, 15, and 30 min following Gd-DTPA instillation. White arrows indicate the Gd-DTPA signals outside the bladder. (**B**) Quantitative assessment of MRI results. Percent (%) change in MRI signal intensity 5, 15, and 30 min following Gd-DTPA instillation (*n* = 3 for each group). (**C**) H&E staining of the native bladder urothelium and reconstructed area (between red lines) in Capsule only group. (**D**) IF staining of Upk3a and Zo-1 for the reconstructed area in Capsule only group (red dashed square box region in C). (**E**) H&E staining of the native bladder urothelium and reconstructed area (between red lines) in Capsule with cells group. (**F**) IF staining of Upk3a and Zo-1 for the reconstructed area in Capsule with cells group (red dashed square box region in E). Scale bar: 1 mm (C, E), 100 μm (D, F)

The primary function of urothelium is to generate a robust barrier, which is maintained through tight junctions that across the superficial umbrella cells and uroplakin proteins at the apical surface of asymmetric unit membrane (AUM) plaques [14, 31]. To assess the barrier function of de novo mucosal tissue from KRT5^high^ TP63-expressing basal cells, we implanted 2 different tissue flaps to 6 rabbits and made 3 sham-operated rabbits as Control group. One month after the surgery, all rabbits survived and there was no serious complication appeared during the experiment. Subsequently, CE-MRI was conducted to detect bladder permeability in vivo, which has been validated to be a reliable way to evaluate the barrier function of bladder mucosa [32, 33]. MRI images were obtained before and over 30 min after intravesical instillation of MRI contrast agent Gd-DTPA, and the permeability of urothelium was assessed by calculating the MRI signal intensity outside the bladder around implanted segments (Fig. 7A). In Control group, there was no leakage of the Gd-DTPA contrast agent before or after instillation of Gd-DTPA. At 5 min after Gd-DTPA instillation, Gd-DTPA signals were observed outside the bladder around implanted segments in Capsule only group, and continued to diffuse and increase from 5 to 30 min. In Capsule with cells group, no significantly visible Gd-DTPA signal was detected outside the bladder which is consistent with the Control group. Quantitative assessment of MRI signals illustrated that there was a sustained increase of MRI signal intensity for the Capsule only group and was significantly higher than that obtained from Capsule with cells group and Control group (Fig. 7B). Lastly, rabbits were sacrificed and gross examination found that the lumen surface of reconstructed area in Capsule only group bladder shrunk towards the flap center and 2 of them with stone formation, in contrast, all rabbits in the Capsule with cells group demonstrated a smooth luminal surface without signs of stone formation (Fig. S5). Furthermore, histological staining showed partial urothelial coverage in the reconstructed area in Capsule only group (Fig. 7C and D), whereas, the Capsule with cells group exhibited a multi-layered urothelium lining the lumen surface of reconstructed area that was similar with the adjacent native urothelium (Fig. 7E). The reconstructed area in the Capsule with cells group expressed pronounced immunoreaction with uroplakin protein Upk3a and tight junction protein zonula occludens-1 (Zo-1) (Fig. 7F). It indicated that the de novo urothelium from KRT5^high^ TP63-expressing basal cells could keep urothelial integrity and barrier functionality.

Overall, our results manifested that generation of KRT5^high^ TP63-expressing basal cell sheet was feasible, and KRT5^high^ TP63-expressing basal cell sheet could resemble the morphology of native urothelium on the surface of capsule. Importantly, the bioengineered urothelium implemented perfect barrier function when implanted to bladder.

## Discussions

In the present study, we provided unequivocal evidence that KRT5^high^ TP63-expressing basal cells play an essential role as stem/progenitor cells in urothelial regeneration of rabbit. Using BrdU labeling and histological methodology, we demonstrated the cell fate of KRT5^high^ TP63-expressing basal cells toward intermediate and superficial umbrella cell types after full-thickness mucosal resection. Furthermore, scRNA-seq data indicated that bladder urothelial cells can be categorized into five different clusters by gene expression characterization and gene enrichment analysis. The Basal cell_2 cells expressing TP63 and higher KRT5 serve as progenitor/stem cells giving rise to Basal cell_1, as well as Intermediate cell_1, Intermediate cell_2 and Umbrella cells in a linear sequence. Notably, we successfully expanded KRT5^high^ TP63-expressing basal cells with capability of self-renew and differentiation, and applied them as “seed cells” for bioengineered urothelium with proper histological architecture and functional barrier.

Tissue-specific stem cell is a rare population residing in specific tissue that are capable of self-renewal and directional differentiation, forming a subset of lineages within the tissue [34, 35]. In epithelial tissues, tissue-specific stem cell is the principal cell type in the cellular hierarchy for homeostasis maintenance and tissue regeneration after injury [36]. As demonstrated in the field of esophagus and trachea regeneration, autologous stem/progenitor cells have been expanded in vitro and successfully applied to reconstruct corresponding mucosa [37–39]. To the best of our knowledge, this is the first successful attempt to isolate and expand the stem/progenitor cells of bladder urothelium in vitro and use them for urothelial tissue engineering in vivo. Here we show that rabbit urothelial stem/progenitor cells near the basement membrane, characterized by co-expression of p63 and KRT5, can be isolated and expanded in vitro, and cultured KRT5^high^ TP63-expressing basal cells can transcriptionally resemble their in vivo counterparts and differentiate into intermediate and superficial umbrella cells. As a result, autologous KRT5^high^ TP63-expressing basal cells transplantation was able to reconstitute an anatomic phenocopy of the native bladder urothelium with perfect barrier function. The feasibility of large-scale expansion in vitro and competent regenerative capacity make KRT5^high^ TP63-expressing basal cells as the optimal “seed cells” for bioengineered urothelium.

Acellular and cellular scaffolds are two major approaches of tissue regeneration by tissue engineering. Preclinical studies revealed that using these strategies could regenerate bladder wall adequately in rodents [40, 41], however, the outcomes of clinical studies tended to fail. Bladder augmentation with SIS in humans showed abnormal bladder tissue with deficient urothelium, indicating that “seed cells” is indispensable for bioengineered urothelium [42]. The bioengineered bladder from cellular scaffolds also failed to improve bladder functionality and developed obvious fibrosis and underwent shrinkage over time owing to poor vascularization [8, 43]. In our rabbit experiment, capsule vascular bed can function as “hospitable soil” to supply sufficient nutrients and oxygen for KRT5^high^ TP63-expressing basal cells in vivo. The bioengineered KRT5^high^ TP63-expressing basal cell sheets can survive on the surface of capsule and resemble the morphology of native urothelium. Moreover, transferring the vascularized bioengineered urothelium with its own axial vessels to bladder can achieve functional barrier capacity similar to native urothelium. Additionally, KRT5^high^ TP63-expressing basal cell sheets were fabricated using cell sheet technology in this study, which can avoid enzymatic digestion and problems of scaffold materials lie in the immunogenicity, uncontrollable degradation, and acid production in the process of degradation [44, 45]. Furthermore, transplanting different cell sheets onto capsule vascular bed layer by layer may construct a multi-layered architecture, which is adequate for bionic reconstruction of bioengineered bladder. Therefore, it is promising to combine KRT5^high^ TP63-expressing basal cell sheets with neural and muscle components based on capsule vascular bed for developing a bioengineered bladder with proper histological structure and satisfactory features.

On the other hand, the bladder wall and urothelium are main targets for the pharmacological treatment of many bladder disorders associated with urothelial dysfunction. Hence, establishing preclinical experimental models that mimic the human bladder urothelium are indispensable for drug screening. Presently, the majority of research on the urothelium has been conducted using mouse models, however, functional differences between rodent and human bladders raise the possibility that a given drug is challenging to translate to humans [46, 47]. Although different urothelial organoids have been used for drug screening, the relevance of current organoids to urothelium in vivo remains in question because such model systems do not account for a large number of factors, including native bladder urothelium architecture and microenvironment [48–51]. The bioengineered model of this study is a promising strategy to develop biomimetic urothelium from human stem/progenitor cells in immunodeficient rabbits [52, 53]. By our strategy, the bioengineered urothelium would be more closely mimic their natural habitats within the human body providing a powerful in vivo model for pharmacology and toxicology experiments. Additionally, several bladder diseases, such as recurrent urinary tract infection, interstitial cystitis/ painful bladder syndrome (IC/PBS), radiation cystitis and bladder cancer, are thought to result from dysfunctional response of stem/progenitor cells to urothelium injury and inadequate repair of urothelium integrity [13, 54–56]. Thus, bioengineered urothelium from stem/progenitor cells of diseased bladder based on the technologies described herein would recapitulate the corresponding human disease and serve as valuable preclinical models for pathophysiological research and therapeutic development on different bladder diseases.

Transformation-related protein 63 (p63), a p53 family member, is proposed as a common stem/progenitor cell marker of several epithelial tissues [57–59], which plays a critical role in urothelium development and regeneration, suggesting that it is a key marker of putative stem/progenitor cells in the urothelium [60, 61]. Under immunohistochemical analysis, we found that p63 was positively stained in mostly intermediate and basal cells of normal rabbit urothelium and all Krt5 positive basal cells coexpression with p63 but not vice versa. Unlike the discrete distribution of Krt5 positive basal cells in rabbit, the basal layer of murine or human urothelium comprises a homogenous population of Krt5 positive cells, however, the scRNA-seq data suggest that their basal cells are also heterogeneity [62, 63]. Thus, an incorporation of more exclusive marker into Krt5/p63 panel may be desired to identify the most potent subpopulation in human urothelium. On the other hand, controversy over the cellular lineage hierarchy within the urothelium still exists, and both linear and nonlinear models of urothelial regeneration have been proposed. The linear model indicates that urothelial stem/progenitor cells locate in the basal cell populations, likely the KRT5 positive basal cells [16, 17, 19, 64]. In contrast to the linear model, the nonlinear model suggests that urothelial stem/progenitor cells are thought to reside within both the intermediate and the basal cell populations and are capable of self-renewal and/or give rise to umbrella cells [18, 20, 60]. Here the scRNA-seq data demonstrated that urothelial differentiation and regeneration occur in a linear fashion, with a single origin of basal stem/progenitor cell progressively differentiating into intermediate and superficial umbrella cells, which is consistent with the lineage hierarchy of skin epidermis and esophageal epithelium [65–67]. Regards to the nonlinear model, one possible explanation is that the incomplete mucosa injury models were used in these experiments.

Our scRNA-seq data revealed that KRT5^high^ TP63-expressing basal cells are enriched in pathways critical for epithelial proliferation and regeneration, including the PI3K-Akt and HIF-1 signaling pathways (Fig. 4G). The PI3K-Akt pathway, known to regulate cell survival, growth, and differentiation in epithelial stem cells, likely supports the proliferative burst of basal cells during injury response [68]. HIF-1 signaling, which is activated under hypoxic conditions, may facilitate metabolic adaptation during tissue repair, as observed in other regenerative contexts. While not explicitly explored here, canonical pathways such as Wnt and Notch—key regulators of epithelial stem cell maintenance and lineage commitment in skin, intestine, and bladder are hypothesized to further orchestrate basal cell activation and differentiation. For instance, Wnt signaling has been implicated in urothelial repair following bacterial injury [19], and Notch is critical for balancing progenitor self-renewal and differentiation in stratified epithelia [55]. Future spatial and temporal transcriptomic analyses will clarify the dynamic interplay of these pathways during regeneration.

The anatomical and physiological similarities between rabbit and human bladders—including multi-layered urothelium and comparable regenerative dynamics—support the translational relevance of our findings. However, several challenges must be addressed before clinical application. First, scalability: while autologous KRT5^high^ TP63-expressing basal cells circumvent immunogenicity, large-scale expansion under Good Manufacturing Practice (GMP) standards requires optimization to ensure genetic stability and differentiation fidelity. Second, microenvironmental factors: the capsule vascular bed strategy, though effective in rabbits, must be adapted to human vascular anatomy and validated for long-term functionality. Third, safety: rigorous preclinical studies are needed to exclude tumorigenic risks, particularly given the proliferative nature of basal cells. Ethical considerations, such as the use of autologous versus allogeneic cells and compliance with regulatory frameworks for engineered tissues, further underscore the complexity of translation.

## Conclusion

In conclusion, our study reveals that KRT5^high^ TP63-expressing basal cell, serving as putative stem/progenitor cells in homeostatic maintenance and regeneration of the mucosa following injury, could be the optimal “seed cells” for bioengineered urothelium. Importantly, the strategy of urothelium engineering described herein would provide numerous applications in near future. Combining KRT5^high^ TP63-expressing basal cell sheets with neural and muscle components layer by layer on capsule vascular bed may construct a multi-layered architecture with proper histological structure and satisfactory features, which is a promising alternative to gastrointestinal tissue for bladder regeneration. Moreover, the bioengineered urothelium from KRT5^high^ TP63-expressing basal cells of normal or diseased human bladder would be a versatile and powerful tool for drug screening and the identification of novel therapeutic targets on different bladder diseases.

## Electronic supplementary material

Below is the link to the electronic supplementary material.

Supplementary Material 1

## Data Availability

The raw data files of RNA-seq have been deposited in the Gene Expression Omnibus GEO data repository under accession code GSE297503. All other data supporting the findings of this study are available from the corresponding author upon request (chenfang01@sjtu.edu.cn).
