# Supplementary material for: KRT5high TP63-expressing urothelial basal cells act as a driver to bladder urothelium regeneration in rabbit
Source: Stem Cell Res Ther. 2025 Jun 7;16:296. doi: 10.1186/s13287-025-04417-z (PMC12145601; doi:10.1186/s13287-025-04417-z)
Supplement: Supplementary file 1 — Supplementary Material 1 [file 13287_2025_4417_MOESM1_ESM.docx]

**Supplementary Figures**

**Fig. S1.** **Focal mucosal resection injury model in rabbit bladder.** (**A**) General view of normal rabbit bladder mucosa. (**B**) Resection of focal bladder mucosa from the bladder.

**Fig. S2. Schematic view of rabbit urothelium layers.**

**Fig. S3. Histological observations of normal** **bladder urothelium.** H&E staining and IF staining of normal murine (**A**) and rabbit (**B**) urothelium. White dashed lines indicate the epithelial-mesenchymal boundary. Scale bar: 200μm (H&E staining), 40 µm (IF staining).

**Fig. S4. Overview of single cells derived from rabbit bladder wall.** (**A**) Violin plots showing the quality control of single-cell data. (**B**) Violin plots show marker genes in different clusters identified. (**C**) UMAP visualization of all cells from bladder urothelium under normal and injured conditions and cells are colored by different cell types.

**Fig. S5. Macroscopic view of the lumen surface of implanted segment in bladder.** (**A**) The lumen surface of reconstructed area in Capsule only group. (**B**) The lumen surface of reconstructed area in Capsule with cells group.
